# Genetic and Pathological Insights into the rs7216389 Polymorphism in Gasdermin B and Its Association with Childhood Asthma

**DOI:** 10.15190/d.2024.15

**Published:** 2024-12-31

**Authors:** Qudsia Umaira Khan, Afreen Banu, Ismail Mazhar, Aimen Binte Asif, Aan Waseem

**Affiliations:** ^1^Department of Physiology, Lincoln University College, Malaysia; ^2^Department of Microbiology and Parasitology, Lincoln University College, Malaysia; ^3^Department of Medicine, CMH Lahore Medical College and Institute of Dentistry, Lahore, Pakistan; ^4^Learning Alliance, Lahore, Pakistan

**Keywords:** Childhood asthma, Asthma pathogenesis, Gasdermin B, GSDMB, rs7216389 variant, 17q21 locus.

## Abstract

This review provides an overview of genetic and pathological mechanisms associated with childhood asthma, focusing on the Gasdermin B (GSDMB) gene variant rs7216389. Accordingly, asthma is outlined as the most common chronic disease in children, with increased incidence in the worldwide community, critically important complications, and mortality related to severe manifestations, primarily exacerbations. The review provides a clinical definition of asthma exacerbation, briefly goes into the cost aspects, and explains the features of pediatric asthma compared to adult-onset asthma. It recognizes the influence of genetic factors such as single nucleotide polymorphisms at the 17q21 locus concerning asthma and its severe attacks while stressing the need to understand those genetic factors that could be potential targets for treatment. The review also stresses the difficulties in implementing the discovery in the clinic, and the potential of additional research dedicated to unveiling the relationship between genetic risk factors, environmental exposures, and immune reactions in the pathological process of childhood asthma. To this end, the current work should be viewed as an attempt to provide a broad overview of asthma pathogenesis and contribute to the development of novel hypotheses and therapeutic approaches in future studies.

## SUMMARY

1.Introduction

2. Clinical Implications of rs7216389 Polymorphism in Childhood Asthma

3. Differential Association of GSDMB with Adult and Childhood Asthma

4. Association of GSDMB with Inflammatory and Immune Responses

5. rs7216389 Polymorphism and Biological Implications

6. Gasdermin B (GSDMB) Gene: Structure and Function

7. Expression Patterns and Regulation of GSDMB in Different Cell Types

8. Genetic and Pathological Factors Influencing Childhood Asthma

9. Conclusion

## 1. Introduction

Asthma is one of the diseases that affect children most frequently and can be considered the most widespread chronic disease in childhood ^1^. Current World Health Organization (WHO) statistics indicate that the incidence of asthma is on the rise across the world^2^. A recent study reported that out of the total expenditure on asthma, eighty-seven percent is spent on acute care, emergency departments, and hospitalizations ^3^. Global Initiative for Asthma (GINA) in this respect defines exacerbations as any period when the patient’s condition is poor enough to warrant a change in management ^4^. These episodes are characterized by an augmentation of the signs that include shortness of breath, cough, wheezing, chest oppression or tightness, and a reduction in the FEV1/FVC ratio ^5^. The condition is characterized by airway inflammation and edema, wheezing, exercise-induced broncho hyperresponsiveness, and increased levels of IgE against inhaled allergens ^6^. However, even today asthma remains a significant global health challenge in the world and still there are numerous issues regarding the molecular mechanism of asthma and possible cure. These are usually characterized specifically by obstruction, wheezing, broncho hyperresponsiveness, and also the increase in the levels of IgE antibodies as the reaction to inhaled allergens ^6^. Most of the features and effects of asthma appear to correlate with the age and gender of the patients even though they are not specifically stated. For example, in children, the disease is predominantly atopic and associated with allergen exposure and shows increased levels of IgE along with a marked reduction of FEV1/FVC ratio and a marked increase in bronchial responsiveness ^6^.

Adult-onset asthma is more common in women than in males and is less likely to be linked to allergens ^4^. Adult asthma is typically difficult to treat and has enduring resistance to effective traditional therapies for childhood asthma ^7^. Tobacco use and exposure to indoor and outdoor air pollution as well as occupational contaminants are the main risk factors for asthma ^8^. Two hundred fifty-one million people worldwide suffer from COPD, whereas three hundred thirty-nine million people worldwide suffer from asthma, and around 1000 people die from asthma-related causes every day ^9^. Around 90% of fatalities in low- and middle-income countries are attributed to COPD, and by 2030, COPD and associated illnesses are predicted to account for 4.5 million deaths worldwide ^10^. COPD is a leading cause of death worldwide. In Pakistan, 7.5 million adults and 15 million children have asthma ^11,12^. According to estimates, 2.1% and 4.3% of Pakistanis suffer from COPD and asthma, respectively ^12^. One-fourth of patients in primary healthcare (PHC) facilities in Pakistan suffer from asthma or COPD, two serious respiratory conditions ^11^.

GWASs have revealed more than 150 genetic markers associated with asthma, and these studies have also provided additional details about the causative agents of asthma in adults and children ^13^. Major SNPs involved in childhood asthma are included in Table 1. Some studies have been conducted to endeavor to identify a link between the rs7216389 polymorphism, situated in 17q21 in the ORMDL3 gene, with childhood asthma. Several studies conducted in connection with research indicate that this particular SNP poses an asthma risk, especially among children and youths ^14,15^. They found that T-cells and airway epithelium play a major role in childhood-onset asthma, and that risk loci and gene tissue specificity differ between childhood and adult-onset asthma ^16^. The heaviest supported locus is a 1. Chromosomal region 17q12-q21 genes are involved in the pathogenesis of the 6-Mb area associated with childhood-onset asthma and were initially described four years ago^15^. Similar to the GWASs, most genetics studies on 17q12-q21 and asthma have been done on populations of European origin, despite the existence of other populations, such as Asians with evident ancestry in this region ^17^.

Several analyses incorporating various ethnic populations, such as African Americans, Asian Americans, and multiethnic samples, revealed that 17q12-q21 SNPs remain significantly associated with asthma ^18,19^. These two regions are, therefore, linked due to the overload of associations with childhood-onset asthma, and it has been difficult to determine the variations and genes in the region that trigger the risks of developing asthma ^5^. Thus, the objective of the present literature review is to assess the role of pathological and genetic factors in childhood asthma, with a focus on the rs7216389 polymorphism of the GSDMB gene. The reason for this review is to provide a definition and clarification on the GSDMB gene, and how it relates to immunological and inflammatory processes and to consider the reasons for different impacts it has on adult and pediatric asthma. Furthermore, the review aims to investigate the biological impact of the identified polymorphism rs7216389 regarding childhood asthma development and progression and GSDMB frequency in various types of cells as well as gene regulation mechanisms. In this detailed study, the review seeks to better understand how the genetic variations in GSDMB affect the predisposition to asthma, which may be important to the next studies and treatment plans.

**Table 1:** Major SNPs involved in childhood asthma

SNPs	Mechanism
ORMDL3	Regulates remodelling genes, metalloproteases, sphingolipids and chemokines.Suppresses the ATF6α branch of the unfolded protein response (UPR) which regulates IL-6 and SERCA2b.
GSDMB	Regulates cell differentiation, cell cycling, and cell death.2. Controls TGF-β1 and 5-LO which are well established to be involved in the pathogenesis of asthma.
	

## 2. Clinical Implications of rs7216389 Polymorphism in Childhood Asthma

ORMDL3, which is modulated by the SNP rs7216389, is involved in the control and maintenance of immune reactions together with inflammation ^7^. Changes in this gene have been reported to influence the regulation of ORMDL 3 plays a role in calcium signaling and inflammation and could be implicated in asthma development and progression ^20^. The subgroup analysis of the studies has supported the fact that the link between rs7216389 and asthma is stronger in children, thus proving the hypothesis, that genetic factors perform a larger role in pediatric asthma than in the differentiated forms of adult-onset asthma ^21^. A meta-analysis of 55 studies, determined that the T allele of the rs7216389 gene is off-risk for childhood asthma. A total of 18 polymorphisms were identified, of which, 9 polymorphisms were associated with asthma risk in overall populations: (IL-13 +2044 G/A, ADAM33 F+1, IL-4 -590C/T, ADAM33 T2, ADAM33 ST+4, ADAM33 T1, ORMDL3 rs7216389, VDR TaqI, and VDR FokI) ^7^. More exactingly, those children in the TT or TC genotype category face a higher risk than the individuals in the CC genotype group ^7^.

**Figure 1:**
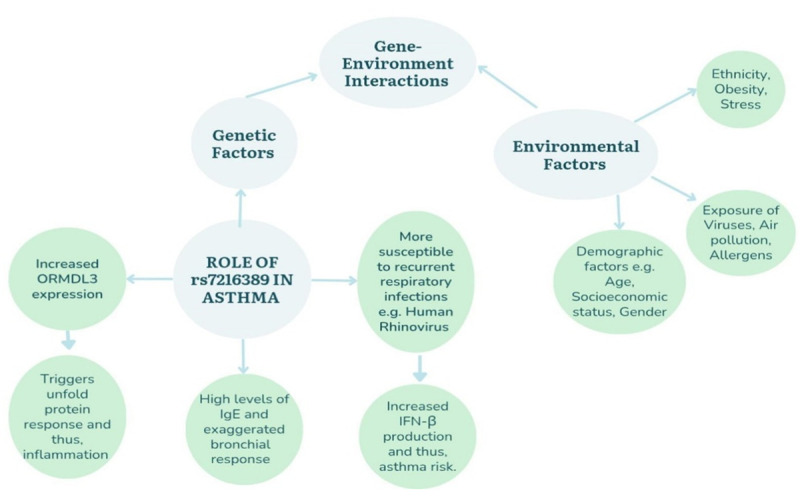
Factors influencing childhood asthma

Meta-analyses of 10 observational research studies put together, reported that children with TT or TC genotypes at the locus of rs7216389 were at a decidedly higher risk of developing asthma than their counterparts with the CC genotype. By comparing with the control group, there exists a statistically significant disparity in the prevalence of rs7216389 polymorphism in the children with asthma (P<0.00001). Moreover, a significant association was also found in Caucasians and Asians (P<0.00001) with the rs7216389 polymorphism. The most striking finding of enhanced susceptibility was seen in atopic asthma (P<0.00001) ^22^. The association between rs7216389 and asthma risk was particularly pronounced in children, suggesting genetic factors may play a more significant role in paediatric asthma compared to adult forms of the disease ^22^. The rs7216389 also regulates immune responses and inflammation. Variations in the rs7216389 gene can also affect ORMDL3 expression, which is involved in calcium homeostasis and the inflammatory response, potentially contributing to asthma pathogenesis in children (Figure 1) ^22^.

Other studies also pointed to the fact that polymorphisms in GSDMB may affect serum IgE concentrations ^23^. In particular, the changes that make the IgE levels reduced had a rather protective effect on asthma, especially in the children, who inherited the rs7216389 variant ^24^. Another study observed that some of a particular genotyping reduced IgE levels considerably and might thus be linked to the prevention of severe asthma attacks ^23^. Foods that are rich in antioxidant vitamins and omega-3 fatty acids are also recommended for people. Diet has an impact on inflammation and the immune system where genetic risks such as the rs7216389 polymorphism could be buffered by nutritional accompaniments or explanations ^19^. As known for asthmatic children, avoiding contact with other allergens (dust mites, pollen, pet dander, etc.) also decreases the risk of asthma manifestation and attacks in children with the rs7216389 polymorphism^25,26^. One of the reported polymorphisms is IL-13 +2044A/G and IL-13 -1112C/T where there is contradicting information with references to asthma^27^. For example, the research on the IL-13 +2044A/G polymorphism has shown that it was informative of the risk of asthma in Asians as well as Caucasians ^28^. The other genotype that has been described to be linked with asthma is the polymorphism IL-4 -590C/T, especially in children of Chinese origin ^29^. To this end, it has been found that this polymorphism is involved in the regulation of the IgE levels that are so crucial in allergy that accompanies asthma ^3^. IL-13 and IL-4 belong to cytokines that are crucial in Th2 immune response which plays a critical role in asthmatic disease ^30^. It was established that they take part in the expression of eosinophilic inflammation and IgE—which reflects allergic asthma.

## 3. Differential Association of GSDMB with Adult and Childhood Asthma

In ethnically diverse cultures, the GSDMB gene on chromosome 17q21 is linked to childhood-onset asthma ^31^. The ORMDL3 gene had a highly reproducible link with childhood-onset asthma at GWAS, not far from GSDMB ^30^. The endoplasmic reticulum protein encoded by ORMDL3 is implicated in several downstream processes, such as sphingolipid production, calcium channel signaling, the unfolded protein response, and inflammation ^27^. It has previously been discovered that variations in the complex 17q21 area, which impact GSDMB and ORMDL3, determine a child's susceptibility to asthma as well as the development of rhinovirus-induced wheeze in preschoolers ^32^. Researchers also found that children in the subgroup with a history of early childhood human rhinovirus (HRV) wheezing disease were more susceptible to the effects of the 17q21 gene on increased propensity ^33^.

The connection affects the transcription of these genes, leading to increased expression of ORMDL3 and GSDMB and reduced expression of ZPBP2 ^23^. GSDMB and ORMDL3 have the same transcription orientation and are located in different kilobase regions. In the genetic structure, the two genes could have the same promoter region ^25^. In GWASs, it was also discovered that there was a substantial correlation between asthma and GSDMB polymorphisms. There are four distinct splice variants that vary in exons 6 and 7 of the human GSDMB gene, out of the 12 exons in total. 73 GSDMB is extremely tissue-specifically expressed only in the skin and gastrointestinal tract epithelium. The bronchial epithelium expresses GSDMB as well, which might be in charge of airway remodeling. A C-terminal repressor domain and a cytotoxic N-terminal domain are also present in GSDMB. It is a pore-forming effector protein that induces pyroptosis, a lytic pro-inflammatory form of cell death, and membrane permeabilization. Strong evidence suggests that this process involves the activation of caspase-1, caspase-11/4/5, caspase-3/7, and caspase-8.

The cytotoxic domain can infiltrate into cell membranes and produce enormous oligomeric holes by proteolytic cleavage between these two domains, which releases intramolecular inhibition and causes pyroptosis ^34^. A splicing variation known as rs11078928 can impact the pyroptotic activity of the GSDMB protein. This variant involves the deletion of exon 6, which contains 13 amino acids in the crucial N terminus. Natural killer cells and cytotoxic lymphocytes can induce pyroptosis in GSDMB-positive cells ^35^. Pyroptosis is encouraged and GSDMB expression is upregulated by interferon-γ (IFN-γ). The C-terminal domain of only GSDMB inhibits the ability of any other Gasdermin N-domain to bind phosphoinositide on the inner plasma membrane leaflet, and only GSDMB has been demonstrated to bind sulfatides ^36^. The GSDMB genes rs2305479A and rs2305480 T are polymorphisms that may cause aberrant sulfatide transport, which could disrupt the integrity of the epithelial cell barrier and/or encourage inflammatory processes. These variants may also have a significant impact on the expression of other nearby genes, including ORDML3 and GSDMA ^25^. The alleles rs11079829-T and p.298Pro are linked to childhood-onset asthma in all populations that have been investigated ^26^. A study used whole-genome sequencing in African Americans to corroborate the localization of this connection to SNPs in the GSDMB gene. This further revealed that the splice variant rs11078928 is the causative SNP at this locus. Studies have linked some allergy disorders, such as hay fever and allergic rhinitis, to certain conditions ^37,38^. The latter may indicate a role for this gene in epithelial barrier function, a trait shared by allergic rhinitis, atopic dermatitis, and asthma that develops in childhood ^3^. Children with early-life respiratory disorders, exposure to ambient tobacco smoke, illnesses linked to wheezing, and wheezing phenotypes have shown the highest associations of SNPs at this locus. Furthermore, the effects of numerous environmental risks for childhood-onset asthma are also moderated by genotypes at this locus ^19,39^. These include exposure to prenatal and postnatal tobacco smoke, wheezing illnesses associated with rhinovirus and respiratory illnesses in general, farm animals, older siblings, house pets, breastfeeding, and mild exposure ^2,23,38^. Few studies have specifically addressed ORMDL3's predictive function on the likelihood of exacerbations, although it may have a role in airway hyperreactivity, sphingolipid production, and allergic reaction, all of which may be related to its possible contribution to asthma pathogenesis ^23,40^. GSDMB, on the other hand, produced more encouraging outcomes. There is a high link between GSDMB and severe exacerbations in the Copenhagen Prospective Studies on Asthma in Childhood exacerbation cohort (COPSAC exacerbation), which is made up of children between the ages of 2 and 6 who frequently require hospitalization for asthma episodes ^4,38,41^. More recently, it was shown that many SNPs in GSDMB enhance its expression and that this corresponds with exacerbations in longitudinal research including a cohort of teenage and adult patients followed for three years ^23^. In Denmark, was conducted a very modest GWA research (1173 cases and 2522 controls) on a clinically important trait in children ages 2–6 years: repeated asthma exacerbations requiring hospitalization ^42^. SNPs in CDHR3, which codes for the transmembrane protein cadherin-related family member 3, which is expressed in ciliated airway epithelial cells, were linked to this trait. It was demonstrated that CDHR3 mediated the binding of Rhinovirus C, a common cold virus that causes severe respiratory tract infections and exacerbations of asthma in children ^42^. An amino acid alteration in the CDHR3 protein caused by a highly correlated SNP not only facilitated increased viral binding and replication but also controlled the quantity of CDHR3 protein ^42^. Since they express more CDHR3 surface receptors on the airway epithelium when attached to Rhinovirus C, children having this risk genotype at the CDHR3 gene are therefore more vulnerable to Rhinovirus C infection, leading to more severe infections and exacerbations of asthma ^31,34^. Despite the genetic association between GSDMB and asthma, the precise biological role of GSDMB in asthma development remains unclear. This genetic variant has been linked to a phenotype marked by reduced lung inflammation and heightened airway responsiveness ^32^. It was demonstrated to be highly expressed in asthmatic patients' T cells and bronchial epithelial cells ^23^. Research conducted on GSDMB overexpression in human lung epithelial cells in vitro revealed the simultaneous elevation of many genes involved in airway hyperresponsiveness, including TGF-β1, leukotrienes, chemokines, and heat shock proteins ^38^. Furthermore, it was also identified that a notable association existed between GSDMB levels and genes related to the Th1 pathway in the antiviral response, as well as genes associated with the type I and type II IFN pathways, and Major Histocompatibility Complex (MHC) class I molecules ^31^. Hence, the interplay between viral agents and the expression of genes related to antiviral pathways, which are controlled by GSDMB DNA variations, can also influence an individual's susceptibility to exacerbations ^32,33^.

## 4. Association of GSDMB with Inflammatory and Immune Responses

The first asthma susceptibility locus was reported in 2007 and localized on chromosome 17q12–21. 1 of which encodes Orosomucoid-1-like-3 (ORMDL3) and the other, Gasdermin B (GSDMB)^26^. These SNPs were validated in two other samples and showed a very high degree of association with childhood asthma ^23^. Another variant is IL1RL1 associated with asthma in the genome-wide approach and also SNPS at PDE4D is associated with asthma in the genome-wide approach ^28^. Another work attributed this to chromosome 5 at 5q31, IL-13, and RAD50. integrated copy number variation and the HLA-DR/DQ gene at 6p21. 3 ^28^. Other GWAS have put forward other polymorphisms including IL33, IL18R1, SMAD3, IL2RB, and CRB1 using large-scale discovery and replication samples ^2,37^. Added evidence for the fact that ORMDL3, the two major asthma candidate genes at this locus are associated with genotypes at SNPs in the core area of the gene ^25^. Further, it was found that chromosome 17q21, linked to SNP in asthma, upregulated ORMDL3 and GSDMB ^40^. ORMDL3, a protein located in the ER, regulates the following downstream processes which include remodelling genes, metalloproteases, sphingolipids, and chemokines ^28^. Furthermore, ORMDL3 also functions to suppress the ATF6α branch of the unfolded protein response (UPR) that regulates IL-6 and SERCA2b as well as repressing the serine palmitoyl- CoA transferase the enzyme that limits the rate of sphingolipid biosynthesis ^18^. However, adopted from a study, it can be suggested that IL6 and SERCA2b can be specifically involved in the pathogenesis of asthma ^21^. In this way, as the UPR increases, there is a low concentration of Ca2+ in the ER ^22^. It is established that TGF-β1 and 5-LO are involved in the pathogenesis of asthma, and both are controlled by GSDMB ^20^. Additionally, it has been demonstrated in the past that mice that express higher amounts of human GSDMB or human ORMDL3 have an asthma phenotype ^17^. All of these results pointed to a connection between asthma and the 17q12 and q21 loci ^19^. Furthermore, recurrent wheezing, asthma, asthma exacerbation, and exercise provocation test (ECT) were connected to the 17q12-q21 focus in children tracked from early infancy to school age ^31^.

## 5. rs7216389 Polymorphism and Biological Implications

The rs7216389 polymorphism, situated in the 17q21 region, has attracted considerable interest in genetic studies of asthma because of its robust correlation with the condition ^43^. The SNP is located within the coding DNA sequence of the ORMDL3 gene which encodes a protein involved in the regulation of sphingolipid metabolism ^29^. GWAS has established that the 17q21 region, harboring the ORMDL3 gene, is genetically linked to asthma, particularly in children ^1^. Several groups have also tried to replicate the connection between rs7216389 and asthma. The result has been a solid and consistent genetic link that cuts across geographical and ethnic divides ^20^. Investigators have established that those who have acquired the risk allele of rs7216389 can acquire asthma compounded in contrast to those who have never had the gene ^19^. This specific SNP contributes more to childhood-onset asthma, a characteristic closely related to atopy and has a clear genetic predisposition ^44^. Overall, there are some negative shifts in the immunological response related to the presence of the risk allele, which affects the genes ORMDL3 thereby enhancing susceptibility to asthma ^45^. For subjects with risk alleles, there was increased imprinting of ORMDL3 genes which might contribute to worsening asthma hallmarked by chronic inflammation and airway sensitization ^7^.

The relationship between rs7216389 and asthma risk is complex and heterogeneous if the biological processes are taken into consideration ^18^. ORMDL3 is involved in the regulation of the metabolism of sphingolipids which plays a functional role in regulating some aspects of the cell destiny such as apoptosis, proliferation, or immunological reactions ^28^. Impairment of the metabolism of sphingolipids can cause alterations in the immunologic process, specifically the activation of inflammatory processes in asthma. For instance, it has been shown that ORMDL3 is involved in the unfolded protein response, which is a cellular response to stress that leads to changes in the endoplasmic reticulum ^45^. The constant activation of the unfolded protein response (UPR) leads to chronic inflammation, which is inherent in asthmatic conditions ^24^. Since the rs7216389 variant could perhaps affect the development of asthma through this route, studies established the relationship between rs7216389 and other environmental factors studies have established the relationship between rs7216389 and other environmental factors, which are well understood to exacerbate asthma including tobacco smoke and respiratory infections ^1,43^. If an individual possesses the risk allele, then maybe the risk of developing asthma could be higher because they may be more sensitive to different environmental stimuli. This interaction of genes and the environment underlines the meaningfulness of the account of genetic and environmental factors in the development of asthma ^46^.

Besides, the rs7216389 polymorphism has been associated with other allergic and inflammatory disorders such as allergic rhinitis and eczema; both conditions are common among patients with asthma ^18^. It would imply that ORMDL3 might be involved in other immune processes associated with atopic diseases ^18^. Due to the multiple and diverse manifestations of the numerous and varied effects of rs7216389, it is clear that genetic and immunological mechanisms are simultaneous ^29^. Identifying this SNP and its association with asthma has further provided new approaches for creating potential therapeutic interventions ^20^. Therefore, researchers would like to understand how this particular SNP affects the ORMDL3 expression and function in individuals with risk alleles to identify ways to modulate sphingolipid metabolism and ER stress signalling in those individuals ^24,45^. These particular treatments have the capability of reducing the impacts of asthma by either averting the inflammation or minimizing the severity of the inflammation that brings about this disease. While knowing the genetic factors of asthma at a good level, the problem arises in the application of the knowledge about the marker rs7216389 ^46^. Further studies are needed to determine more clearly a definite role of this SNP in each of the above-stated interrelated diseases and the pathways that are affected ^29^.

## 6. Gasdermin B (GSDMB) Gene: Structure and Function

In GWAS, it has been identified that there are two risk genes associated with asthma and these mark the GSDMB and ORMDL3 at chromosome 17q12 ^34^. The GSDMB gene codes for the protein Gasdermin B which is a member of the Gasdermin domain-containing protein family ^31^. Gasdermin B participates in several cellular procedures linked to tumorigenesis and tumor development and these include cell differentiation, cell cycling, and cell death ^37^. GSDMB also known as GSDML can have 411 amino acids and is more differentiated in the human body than other proteins in the GSDMB family ^3^. GSDMB is human-specific, while GSDMA is encoded by the human genome but is systematically absent in both mice and rats. However, there is supposed to be an orthologue of GSDMB expressed in rodents ^31^. In humans, there are six different isoforms for the GSDMB gene by the mechanism of alternative splicing. These variants are present in the human tissues and cells concerning the airway epithelium ^31^. The GSDMB mRNA was predominantly detected in the basal area of the oesophagus and the stomach which are areas of the body that consist of stem cells^33^.

GSDMA, GSDMB, GSDMC, and GSDMD are the four members of the human GSDM family (Table 2)^32^, all of them being transmembrane proteins, and all of them are transmembrane proteins, containing a signal peptide, a transmembrane region, and a hemagglutinin region. The genes for GSDMC and GSDMD are located on chromosome 8 at q24, whereas the genes for GSDMA and GSDMB are located on chromosome 17 at q21 ^30^. Although GSDMB located on chromosome 17q21 has been linked to asthma in many of these GWAS and genetic linkage studies, no other study has previously reported an association between asthma and chromosome 8q24, where GSDMC and GSDMD are located ^47^. A study identified that only GSDMB and maybe GSDMA members of the GSDM family are linked to asthma. The chromosome 17q21 SNP linked to asthma is connected to higher expression of GSDMB and ORMDL3 ^29^. Strong linkage disequilibrium between these SNPs has been shown in several studies, indicating that these genes may be working together to influence the pathophysiology of asthma ^2,23^.

**Table 2:** Members of GSDM protein family

Gene	Locus	Association with asthma
GSDMA	Chromosome 17q21	+/-
GSDMB	Chromosome 17q21	+
GSDMC	Chromosome 8q24	-
GSDMD	Chromosome 8q24	-

## 7. Expression Patterns and Regulation of GSDMB in Different Cell Types

According to recent transcriptome-wide association research conducted on blood and lung samples, GSDMB is the gene most strongly linked to asthma ^48^. According to the Genotype-Tissue Expression (GTEx) database, GSDMB is widely expressed in a variety of tissues and cell types ^49^. The skin, lung, whole blood, and spleen have intermediate levels of GSDMB expression; these tissues are also enriched for the expression of genes at asthma GWAS loci ^50^. These tissues contain a variety of cell types, most likely exhibiting varying levels of GSDMB expression. Numerous cell types, including epithelial, endothelial, and immune cell subsets, express GSDMB ^51^. However, single-cell RNA sequencing in lung tissue, including GTEx, shows that GSDMB expression is confined to a few cells in each cell type ^52^. Therefore, additional investigation is needed to learn how GSDMB is expressed in specific immune cells and lung cells, including lung-resident macrophages and CD4 tissue-resident T cells, as well as airway smooth muscle and epithelial cells ^53^. This is especially important when considering environmental exposures like airway microbes, smoke from tobacco products, and rhinovirus infection. The way that genetic variations linked to asthma affect GSDMB varies according to the tissue ^54^. Increased GSDMB expression and the asthma-risk allele are correlated in all of these organs. Curiously, however, this SNP does not serve as an expression quantitative trait locus for GSDMB in other tissues such as the esophagus, heart, and brain ^55^. This demonstrates how gene regulation at this locus is particular to tissues. The quantity of functional GSDMB protein and the precision with which GSDMB transcripts are spliced have been linked to the asthma risk variant rs11078928 ^56^. Exon 6 is skipped as a result of the non-risk allele rs11078928-C, which also results in reduced synthesis of the GSDMB protein and the full-length transcript ^57^. This indicates that among carriers of the rs11078928-T variation, overexpression of GSDMB protein mediates the asthma risk associated with this gene ^58^.

## 8. Genetic and Pathological Factors Influencing Childhood Asthma

Another important characteristic of the studied subject is that asthma is genetically inherited, its manifestations are seen in successive generations, while in 60% of cases, there is a history of its occurrence in the family ^17^. Association studies help characterize new treatments for this persistent condition and explain procedures connected with the onset of the disease ^59^. A relationship between IgE regulatory genes and asthma-vulnerable genes could exist, as pointed out by the emerging genetic association and susceptibility studies that associate IgE serum levels to asthma vulnerability genes in children and adults ^16^. In contrast, a number of prior genome-wide association studies have demonstrated a correlation between the polymorphisms in Gasdermin A and B and the susceptibility to adult and paediatric asthma in various populations ^60^. Human GSDMA and GSDMB genes are found on chromosome 17 (17q12-21), along with other asthma genes that are strong candidates, including post-GPI attachment to proteins 3 (PGAP3) and ORM1-like gene (ORMDL3) ^13^. Genes in the proximal, core, and distal areas of 17q12-21 have been linked to asthma in another research; the core region, which houses the GSDMB gene, has been linked to the early onset of asthma^61^. It is also connected to wheezing interaction generated by rhinovirus (RV) in early life, which is linked to the development of asthma later on, and pediatric asthma ^62^.

Asthma and allergic rhinitis cause increased serum IgE immunoglobulin antibody levels that is critical for the pathogenesis of allergic disorders ^63^. Several factors of inflammation such as prostaglandins and histamine are released from mast cells in asthma and are linked with the IgE immunoglobulin ^14^. These inflammatory mediators stimulate the production of excessive mucus, which adds supraglottic stenosis to the other causes. Nevertheless, a paper comparing whether asthma was solely responsible for sensitization, revealed that asthmatics were inclined to overproduce IgE when exposed to airborne allergens ^15^. The findings from this study imply that elements of genes might contribute to the regulation of synthesis and manifestation of IgE in asthma, albeit that high risks of developing asthma are not consequent of direct contact with allergens ^15^. Similarly, researchers have brought to light that gen for IgE and gen control of asthma are related in a narrow sense ^4^. Others discovered that there is a co-inheritance pattern between total IgE serum, which is not cognate, and IL-4 at the 5q31 locus. 1. This indicates that it is the IL-4 gene or genes near it that control the synthesis of IgE when there are no allergic antigens ^30^.

The specific object of attention in the present study was a polymorphism within the GSDMB gene, and more particularly in the region of rs7216389, which was found to have an extremely strong link to asthma. The T/C genotype was expressed in 64% of asthmatic children, and the T/T genotype in the rest indicating a gene dose effect in asthma associated phenotype. Further analyses were able to show that rs7216389 is likely to affect immune system regulation, airway epithelial function and inflammatory response hence having a direct impact on asthma pathogenesis. This study supports the fact that rs7216389 has potential to be used to as a marker for asthma with family history and environmental factors including exposure to smoke.

## 9. Conclusion

Asthma is a complex and diverse disease, especially in childhood, and is considered to be the leading chronic disease affecting children all over the world. This is highlighted by the increasing incidence of asthma as documented by WHO surveillance, and the high level of morbidity and mortality that is often witnessed in patients experiencing asthma exacerbation. The review also outlines the clinical significance of exacerbations, which involve worsening signs and symptomatology, and a decline in lung function, that requires a change of therapy. Additionally, the review examines the economic burden of asthma and divulges that acute care visits and hospitalizations add to the cost of medical expenses. The review also describes differences in children's asthma – its atopic nature and its link to allergen exposure compared to adult asthma which is less associated with allergens and affects women more often. In addition to the clinical aspects, the review provides genetic information on asthma, especially on SNPs. These genetic factors have been associated with a heightened risk of asthma and the worsening of this condition, due to the unique SNPs affecting the proteins involved in the process of viral infection and inflammation. The works suggests that decoding these biomarkers could reveal how external signals impact asthma development.
